# Significance of Hereditary Hemochromatosis Gene (*HFE*) Mutations in Chronic Hepatitis C and Hepatocellular Carcinoma Patients in Egypt: A Pilot Study

**DOI:** 10.31557/APJCP.2021.22.9.2837

**Published:** 2021-09

**Authors:** Reham M Dawood, Mai Abd El-Meguid, Walied Elrobe, Ghada M Salum, Naglaa Zayed, Sherief Mousa, Eman Medhat

**Affiliations:** 1 *Department of Microbial Biotechnology, Genetic Engineering Division, National Research Center, Giza, Egypt. *; 2 *Department of Endemic Medicine, Faculty of Medicine, Cairo University, Cairo, Egypt. *

**Keywords:** HFE gene polymorphism, hemochromatosis, hepatitis C, Iron

## Abstract

**Background:**

Hereditary hemochromatosis is a genetic disease defined by enhanced overloading of iron and associated with Chronic Hepatitis C (CHC). This study aims to evaluate the correlation of the *HFE* gene mutations on Egyptian CHC with liver disease progression and the risk of HCC development.

**Methods:**

The HFE mutations (C282Y and H63D) were genotyped on 100 CHC patients and 50 healthy individuals by a hybridization assay. The serum iron content was also measured for all subjects.

**Results:**

A significant elevation of the serum iron, ferritin, and TIBC in HCV-infected patients (p≤0.05). The H63D mutation was detected in 23% of the all studied samples. The serum iron and the H63D heterozygosity were correlated significantly between non-cirrhotic and cirrhotic without HCC patients.

**Conclusion:**

The H63D heterozygosity might have a potential role in iron accumulation. Moreover, HFE mutations did not tend to be associated with an increased risk of HCC in cirrhotic patients.

## Introduction

Infection with hepatitis C virus (HCV) is one of the most predominant causes of chronic liver disease, infecting 71.1 million individuals, about 700,000 deaths worldwide annually (Masavuli et al., 2019). HCV often infects the liver, causing acute and chronic necro-inflammatory damage, including cirrhosis and hepatocellular carcinoma (HCC) (Ringelhan et al., 2017). 

Iron parameters, especially ferritin, are often increased in patients with chronic hepatitis C, indicating a correlation between chronic hepatitis C and iron overload, (Vagu et al., 2013) To date, several different signals have been activated by HCV leading to the formation of reactive oxygen species (ROS) in the liver and promoting liver fibrosis and HCC (Drakesmith and Prentice, 2008; Georgopoulou et al., 2014). Often, HCV changes iron metabolism by reducing hepcidin (iron-regulating hormone) levels (Horl and Schmidt, 2014; Vela, 2018). Hepcidin levels in CHC patients are strongly associated with serum ferritin and the histological iron score (Girelli et al., 2009). It was proved that the HCV polyprotein produced in transgenic mice downregulated the expression of the hepcidin as a consequence of the oxidative stress induced by HCV (Nishina et al., 2008).

Disruption of hepcidin synthesis may contribute to hemochromatosis, hereditary hemochromatosis (HH) is an autosomal recessive syndrome defined by excessive deposition of iron in hepatocytes due to enhanced bowel absorption (Hanson et al., 2001; Procaccini and Kowdley, 2018). Iron overload in all types of HH, results from failure in regulatory pathway for hepcidin. The *HFE* gene is a candidate for inherited hemochromatosis (Bodmer et al., 1997). The *HFE* gene is situated on the short arm of chromosome 6 (van Bokhoven et al., 2011), encodes Human homeostatic iron regulator protein (HFE protein) that controls iron levels in hepatic cells by averting the transferrin receptor from interacting with transferrin as well as controls the production of hepcidin protein (Gao et al., 2009). The HFE protein is made up of 343 amino acid that show substantial resemblance to class Ι molecules of human leucocytes antigen (HLA) (Feder et al., 1996). Hemochromatosis is a result of at least two missense *HFE* gene mutations (C282Y and H63D) (Feder et al., 1996). The C282Y polymorphism, in which a G nucleotide at position 845 of the *HFE* gene is changed to an A nucleotide, resulted in a substitution of cysteine amino acid with a tyrosine residue at amino acid 282. The polymorphism of His 63Asp (H63D), in which a C nucleotide at position 187 of the *HFE* gene is changed to a G nucleotide, results in a replacement of histidine with aspartic acid at amino acid 63 (Pietrangelo, 2006; Sendi and Mehrab-Mohseni, 2012). Patients of inherited hemochromatosis (HH) have a higher chance of developing hepatocellular carcinoma (HCC) relative to non-iron-related chronic liver disease patients (Fracanzani et al., 2001). Interestingly, the increased iron deposition have been associated with poor response to HCV treatment and disease progression (Bartolomei et al., 2011). Recently, an increased incidence of the HFE C282Y heterozygote mutation has been identified in HCC patients and correlated with excessive iron deposition in many organs, indicating a potential role of the HFE C282Y heterozygous state in hepatocarcinogenesis disease (Ye et al., 2016). 

Two other mutations are detected in the* HFE* gene, one of them is (H63D) in which aspartate is substituted for histidine at amino acid position 63 and the other one is (S65C) in which cysteine is substituted for serine at amino acid level 65 (Moysés et al., 2008). 

The role of the H63D and S65C mutations in the iron deposition and the development of HCC remain unclear. However, their association with iron accumulation is observed with C282Y as a compound heterozygote, C282Y/H63D or C282Y/S65C (Blanc et al., 2000; Gochee et al., 2002). It was observed that the iron accumulation had a direct effect on the oxidative stress through the induction of peroxidation of the phospholipid in mitochondria and microsomal membranes. Additionally, the HCV infection showed a strong evidence for the formation of free radicals by activating the enzyme nicotinamide adenine dinucleotide phosphate (NADPH) oxidase (Britton et al., 1990; Thorén et al., 2004; Bloomer and Brown, 2019). It inspired us to determine the occurrence of C282Y and H63D mutations in cirrhotic patients with or without HCC.

The goal of the current study was to explore the correlation between *HFE* gene mutations (C282Y and H63D) and the incidence of HCV-related liver disease as well as the risk of HCC development. Moreover, we aimed to examine the correlation of serum iron profile with the frequency of HFE (C282Y and H63D) mutations in HCV patients with Egyptian ancestry. 

## Materials and Methods

*Subjects*

The current study performed from April 2015 to February 2016 on 150 participants and the enrollment subjects were categorized into 4 groups: 1) Normal individuals (n= 50) had generally normal laboratory test values, no signs of chronic liver disease and no evidence of HBV (HBsAg, HBcAg) or HCV Ab. 2) Patients with non-cirrhotic chronic hepatitis C (n=18) with fibrosis stage <F3. 3) Cirrhotic HCV patients (n=45) without hepatocellular carcinoma. 4) Cirrhotic HCV patients (n=37) with hepatocellular carcinoma. Both patients’ group tested positive for both the anti-HCV antibody and the serum HCV RNA. Child-Pugh Score transient elastography (Fibroscan) tests measured the extent of liver cirrhosis. Patients were assessed with respect to clinical criteria, biochemical data, viral HCV load, histological features and parameters of iron level. The diagnosis of HCC was indicated as focal hepatic lesions (diagnosed by ultrasound imaging) in addition to AFP. All subjects were recruited from department of Endemic medicine (Faculty of Medicine - Cairo University) and the viral hepatitis treatment clinic (Ahmed Maher teaching hospital - National Committee for Control Viral Hepatitis (NCCVH). Exclusion criteria were as follows: thalassemia, diabetes mellitus, skin pigmentation, cardiomyopathy, hypogonadism and arthropathy. According to the Helsinki Declaration 1975, amended in 2008, the experiments were authorized by the National Hepatology & Tropical Medicine Research Institute’s Ethical Review Board (9-2015). Each subject was given informed consent before collecting blood samples.

All parameters of the liver function tests (total bilirubin, direct bilirubin, aspartate aminotransferase (AST), alanine aminotransferase (ALT), total protein, albumin, alkaline phosphatase) were examined. 

*Iron profile*

Evaluation of the iron level for each individual was assessed using biochemical tests. Serum iron (normal for male’s 10–40 µmol/l, female’s 8–40 µmol/l) was evaluated by the ferrozine method. The ferritin level (normal 15–300 ng/ml) was measured by turbidimetry using a Chiron ACS180 automated analyzer (Bayer, USA), and the transferrin (normal 2–4 g/l) was measured by nephelometry (Beckmann Array Analyzer, USA). The measurement of transferrin saturation was calculated as follows: serum iron×70.9/serum transferrin (normal 16–45%).

*HCV determination*

HCV IgG antibodies were tested using third generation enzyme linked immunosorbent assay (Ortho Diagnostics, Raritan, New Jersey, USA), and the HCV-RNA was detected on the patient’s sera by real time PCR (Artus HCV QS RGQ Kit Qiagen, Hilden, Germany). Genotyping of HCV in plasma samples was performed using commercial assays [InnoLiPA HCV II assay, Innogenetics Inc., Alpharetta, GA, USA].

*DNA isolation*

Genomic DNA from peripheral blood leucocytes was extracted using a commercial kit (Promega, MA, USA). The isolated DNA was then stored at -20 ° C until use.

*Amplification of HFE gene*

HFE exon 2 and 4 sequences (exon 2:His63Asp and exon 4:Cys282Tyr) have been amplified with 5’-biotinylated primers in a single, multiplex PCR reaction (Feder et al., 1996; Jeffrey et al., 1999). The PCR amplification mixture composed of 0.4 mM of each primer, 25 mM of MgCl2, 10 mM of each deoxy nucleotide triphosphate (dNTP) (Promega, Madison, WI, USA), 0.5 U of hot start Taq DNA polymerase (Promega, Madison, WI, USA), 5X reaction buffer, 50 ng of genomic DNA and, distilled water to a final volume of (50 µl). Two negative controls (in which water replaced the DNA sample as a check for contamination) were incorporated into each run. PCR reactions were performed in a thermocycler machine (Applied Biosystems, USA). A thermo-cycling condition was as follows: one cycle (2 min at 95°C) accompanied by 35 cycles (94°C for 30 s; 60°C for 30 s; 72°C for 30 s) followed by one cycle final extension (68°C for 7 min). 

*HFE mutation identification*

Mutations of Haemochromatosis were detected using the Haemochromatosis Strip Assay A^®^ (Vienna Lab, Labor diagnostika, GmbH, Vienna, Austria) (Daher et al., 2011), in which the amplification products were hybridized to a test strip providing a parallel array of allele-specific probes according to the manufacturer’s protocols. Briefly, in separate lanes of a thin-walled plastic incubation tray (Bio-Rad, Hercules, CA), ten ml of PCR product was mixed with the denaturing solution (400 mM NaOH, 10 mM EDTA) and incubated at room temperature for 5 min. Then, the alkaline reaction was neutralized with sodium phosphate solution, pH 7 and the reaction mixture was applied on the test strip for 30 min at 45°C. Bonded PCR fragments were found after three rigorous washings at 45°C using a streptavidin-alkaline phosphatase conjugate and color substrates (NBT / BCIP). After positive reaction, a violet staining was noticeable at room temperature after 15 min of incubation, the color signals on the test strip showed the presence of a wild type and/or a mutation (s).

*Statistical analysis*

All statistical calculations were done using SPSS (statistical package for the social science version 26.00) statistical program at 0.05, 0.01 and 0.001 level of probability, Qualitative (categorical) data were presented by frequency and percentage was done using chi square. Quantitative data with non-parametric distribution were done using Analysis of variance Mann Whitney test to comparison between two groups, Kruskal–Wallis test were used for more than two independent groups a relationship between independent and dependent variables were determined statistically using regression analysis. In the present work, Linear Regression analysis was carried out using the stepwise strategy to find out independent factors affecting serum iron. The confidence interval was set to 95% and the margin of error accepted was set to 5%. The p-value was considered non-significant (NS) at the level of > 0.05, ** significant at the level of < 0.05 and ***; highly significant at the level of < 0.001. 

To identify the variation between two independent group means using the Mann Whitney test, an a priori power analysis was performed using G*Power 3 (Faul et al., 2007).

## Results

*Sample description*

This study involved one hundred chronic liver disease patients with HCV infection, and fifty controls. They were classified into four groups; first group: Normal participants; second group: Non cirrhotic HCV patients; third group: cirrhotic HCV patients without HCC; and fourth group: cirrhotic HCV patients with HCC. Table 1 summarizes clinical and demographic features of the examined groups. 

Hemoglobin (Hb), albumin, and direct bilirubin were significantly lower and Serum Alfa fetoprotein (AFP) and total bilirubin were significantly higher in HCC patients than those in other groups. In the cirrhotic patients with HCC, results revealed that Alanine aminotransferase (ALT), Aspartate aminotransferase (AST) and White blood cells (WBC) were significantly high while platelet counts declined markedly relative to the control group. On the other hand, significant differences were observed in International normalized ratio (INR) and Prothrombin concentration (PC) between cirrhotic patients with HCC, non-cirrhotic patients and controls as well. However, there were no significant differences in age, gender and creatinine among the studied groups.

*Iron profile*

All participants were evaluated for total iron, serum transferrin and serum ferritin, and total iron binding capacity (TIBC). It was observed that the serum iron, the serum ferritin and TIBC were significantly elevated in the cirrhotic HCC patients when compared to control group. There was no sharp distinction between the non-cirrhotic group, the cirrhotic group, and the HCC group in Iron and Ferritin, but a surprising distinction exists relative to the control group as described in Table 2.

**Figure 1:**
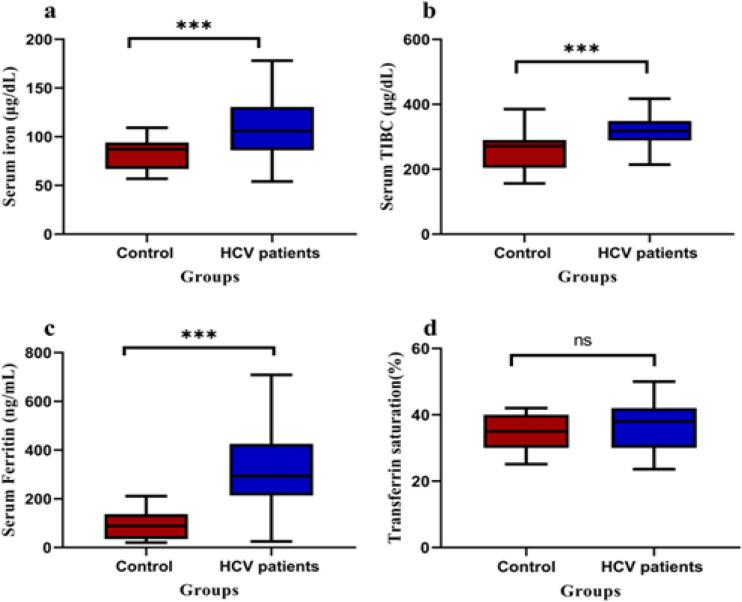
Boxplot Shows Comparison between Control and HCV Patients Regarding: a) Serum Iron, b) Serum TIBC, c) Serum Ferritin and d) transferrin saturation. In control group (n=50) and HCV patients (n=100). The statistical comparisons were done by using Mann–Whitney test., the data was displayed as median (p < 0.001).

Moreover, in all chronic HCV patients, serum iron, serum TIBC and serum ferritin were significantly higher than those individuals in control group (p≤ 0.001), while transferrin saturation didn’t display significant difference among the enrolled groups (p≤ 0.088) as illustrated in Figure 1.

*Frequency of C282Y and H63D genotypes in Egyptian population*

The distribution of the Cys282Tyr and His63Asp mutations of *HFE* gene are as follow, in control group (n=50), HCV patients without HCC (n=63) and HCC patients (n=37) as shown in Table 3. 

The Cys282Tyr mutation of *HFE* gene (genotypes AA, GA) has not been detected in the all studied groups. While in the case of the H63D mutation, the genotypes GG, GC of HFE were detected in 36% of healthy individuals (4% homozygous and 32% heterozygous), 17.5 % in patients without HCC (0% homozygous and 17.5 % heterozygous), and 16.2% in HCC patients (0% homozygous and 16.2% heterozygous).

The C allele frequency for the (His63Asp) mutation was detected in 80 % of healthy group, 91.3% in HCV patients without HCC and 91.9% in HCC patients. 

*The relation between H63D polymorphism and iron markers*

Our findings revealed a significant increase in serum iron in the control group of CC genotypes relative to CG genotype. While in the non-cirrhotic HCV patients, the serum iron is elevated significantly in patients who had CG genotype. However, no significant changes in serum iron were observed among the different mutations carriers in the HCC patients. In addition, no remarkable statistical variations in TIBC, serum ferritin, and transferrin saturation have been detected among all the studied groups. The association between serum iron markers and the H63D mutation is shown in Table 4.

*Logistic regression analysis*

Logistic regression analysis was used to calculate the probability of increasing iron serum using the logit (P) equation, for all the differentially distributed variables (H63D mutation, Chronic HCV, and H63D mutation + Chronic HCV). Regression analysis indicated that HCV infection alone or accompanied by H63D mutation can influence significantly the serum iron elevation. While, the mutation H63D alone might reduce the amount of serum iron as depicted in Table 5. 

**Table 1:** Demographic and Laboratory Data of the Studied Subjects

Clinical and biochemical parameters		Control group	Non cirrhotic group	Cirrhotic group without HCC	Cirrhotic group with HCC	P value
		(N=50)	(N=18)	(N=45)	(N=37)	
Age (years)	Median (IQR)	50 a	50 a	52 a	52 a	0.121ns ‡
		(44.75-55.00)	(48.75-53.25)	(47.00-56.00)	(49.00-59.50)	
Sex (n, %)	Male	28 (56%)	14 (77.8%)	28 (62.2%)	27 (73%)	
	Female	22(44%)	4 (22.2%)	17 (37.8%)	10 (27%)	0.237 ns*
Hb (gm/dL) M:13.5-17.5 F:12.0-15.0	Median (IQR)	13.40 a	13.80 a	14.00 a	11.90 b	<0.001 HS‡
		(13.00-14.00)	(11.92-15.50)	(12.95-14.55)	(11.15-12.65)	
					
PLT=150-450 (x10^3^/mL)	Median (IQR)	272.50 a	197.50 ab	156 bc	110.00 c	<0.001 HS‡
		(210.00-325.00)	(156.75-270.00)	(143.00-207.00)	(83.00-162.00)	
WBC= 4-11 (x10^3^/mL)	Median (IQR)	6.35 a	6.75 a	4.7 b	6.2 a	<0.001 HS‡
		(5.30-7.90)	(4.80-8.20)	(4.00-5.25)	(5.00-8.00)	
ALT= 7-56 (IU/L)	Median (IQR)	33 b	37.50 a	49 a	57 a	<0.001 HS‡
		(27.00-42.00)	(28.75-64.75)	(38.50-65.00)	(46.00-67.00)	
AST= 10-40 (IU/L)	Median (IQR)	25.50 c	44.50 b	45.00 b	68.00 a	<0.001 HS‡
		(19.50-34.00)	(31.00-68.75)	(39.00-67.00)	(42.50-92.50)	
Albumin= 3.5-5.5 (gm/dL)	Median (IQR)	3.6 b	4.25 a	3.90 a	2.7 c	<0.001 HS‡
		(3.4-4.5)	(3.97-4.40)	(3.5-4.35)	(3.6-3.6)	
Total Bilirubin= 0.1-1.2 (mg/dL)	Median (IQR)	0.9 b	0.65 b	0.8 b	1.6 a	<0.001 HS‡
		(0.7-1.00)	(0.5-1.00)	(0.3-0.95)	(0.9-2.00)	
Direct Bilirubin < 0.3 (mg/dL)	Median (IQR)	0.4 b	0.2 b	0.5 b	0.7 a	0.018 S‡
		(0.3-0.4)	(0.1-0.5)	(0.2-0.7)	(0.2-0.5)	
AFP <10 (ng/mL)	Median (IQR)	--	5.88 b	4.6 b	155 a	<0.001 HS‡
			(2.77-18.17)	(2.6-10.7)	(66.8-887.00)	
PC= 70-120 (%)	Median (IQR)	107 a	96.50 b	90 b	75 c	<0.001 HS‡
		(100-119)	(89-100)	(85-98)	(66-80)	
INR ≤1.1	Median (IQR)	1.00 b	1.00 b	1.10 a	1.20 a	<0.001 HS‡
		(0.90-1.035)	(1.00-1.025)	(1.08-1.20)	(1.10-1.4)	
Creatinine= 0.6-1.2 (mg/dL)	Median (IQR)	1.00 a	0.75 b	0.90 ab	1.00 a	<0.001 HS‡
		(0.80-1.010)	(0.70-0.90)	(0.80-1.00)	(0.80-1.1)	
		A	--	37 (82.2%)	8 (21.6%)	<0.001 HS*
Child grades		B	--	8 (17.8%)	27 (73.0%)	
		C	--	0 (0.0%)	2 (5.4%)	
Fibrosis stage		F1	8 (44.44%)	--	--	<0.001 HS*
		F2	10 (55.56%)	-	--	
		F3	--	14 (31.11%)	--	
		F4	--	31 (68.89%)	37 (100.0%)	
MELD score		Median (IQR)	--	7	10	<0.001 HS*
				(6.00-8.00)	(9.00-11.00)	

HB, hemoglobin; WBC, white blood cell; ALT, alanine transaminase; AST, aspartate transaminase; AFP, Alfa fetoprotein; PC, Prothrombin concentration; INR, International normalized ratio; IQR, Interquartile Range; N, number of cases. Statistical comparison was performed using *Chi-square test; ‡ Kruskal Wallis test. According to the Duncan test, the different letters (a, b, c) in the same row are significantly different at p value 0.05. ***; Highly significant at p value <0.001, ns; non-significant at p value >0.05. Results are represented as median (Interquartile Range).

**Table 2:** Assessment of Iron Profile in Control Group, Chronic Hepatitis C and HCC Patients

Variables		Control group	Non cirrhotic group	Cirrhotic group without HCC	Cirrhotic group with HCC	P value
		(N=50)	(N=18)	(N=45)	(N=37)	
Serum Iron=60-170 (μg/dL)	Median	87.20 c	114.50 ab	93.00 bc	114.70 a	<0.001 HS‡
	(IQR)	(66.90-94.04)	(91.25-133.00)	(74.00-108.00)	(93.00-157.90)	
TIBC= 240 – 450 (μg/dL)	Median	271.00 b	315 a	337 a	318.70 a	<0.001 HS‡
	(IQR)	(204-290.42)	(280.75-321)	(297-348)	(284.7-371.50)	
Serum Ferritin = M:12-300, F:12-150 (ng/mL)	Median	88.70 c	323 a	307.00 a	196.60 b	<0.001 HS‡
	(IQR)	(35.17-137.25)	(267.75-422.75)	(272.50-439.00)	(71.40-304.40)	

Statistical comparison was performed using ‡ Kruskal Wallis test According to the Duncan test, the different letters (a, b, c) in the same row are significantly different at p value 0.05. ***; Highly significant at p value <0.001, ns; non-significant at p value >0.05. Results are represented as median (Interquartile Range).

**Table 3:** Genotyping and Allelic Frequencies OF *HFE* Gene in Control, Chronic Hepatitis C and HCC Patients

Variables		Control group (N=50)	HCV patients without HCC (N=63)	HCV patients with HCC (N=37)	P value
C282Y	Wild (GG)	50 (100%)	63 (100%)	37 (100%)	----------
H63D	Wild (CC)	32 (64%)	52 (82.5%)	31 (83.8%)	0.061 ns *
type	Hetero (CG)	16 (32%)	11 (17.5%)	6 (16.2%)	
	Homo (GG)	2 (4%)	0 (0%)	0 (0.0%)	
H63D mutation	Wild= (115/150) 77%	32 (64%)	52 (82.5%)	31 (83.8%)	0.034 S*
	Mutant= (35/150) 23%	18 (36%)	11 (17.5%)	6 (16.2%)	
		Control group (N=100)	HCV patients without HCC (N=126)	HCV patients with HCC (N=74)	P value
H63D alleles	C	80 (80%)	115 (91.3%)	68 (91.9%)	0.017 S *
	G	20 (20%)	11 (8.7%)	6 (8.1%)	

Statistical comparison was performed using *Chi-square test (P-value >0.05, Non-significant (NS); P-value <0.05, Significant (S)).

**Table 4:** Relationship between the H63D Mutation and Iron Profile

	Control n= 50			HCV non HCC patients n=63			HCV with HCC patients		
	Wild H63D	Mutant H63D	P value+	Wild H63D	Mutant H63D	P value+	Wild H63D	Mutant H63D	P value+
	N=32	N=18		N=52	N=11		N=31	N=6	
Iron= 60-170 (μg/dL)	91.4	71.8	<0.001 HS	100.5	164	<0.001 HS‡	100	111.1	<0.001 HS
	(75.72-97.30)	(59.80-85.62)		(83.00-127.50)	(148.00-167.00)		(90.00-102.00)	(104.17-122.17)	
TIBC= 240 – 450 (μg/dL)	271	241	0.976 ns	310	320.58	0.257 ns	350	326.8	0.082 ns
	(197-291.27)	(204-287.85)		(290.00-317.25)	(299.00-357.00)		(349.00-360.00)	(307.85-355.00)	
Transferrin saturation= 25-35%	35.65	33.5	0.117 ns	39	40	0.531 ns	33	35.85	0.470 ns
	(30.00-42.00)	(30.75-37.25)		(36.70-42.00)	(33.00-51.00)		(26.00-41.00)	(34.80-37.05)	
Ferritin =M:12-300, F: 12.150 (ng/mL)	88.7	63.85	0.253 ns	313	292	0.520 ns	210.80 (80.40-304.90)	80.85	0.076 ns
	(66.30-142.15)	(24.10-133.07)		(276.25-437)	(262-439)			(25.30-275.03)	

Results are represented as median (Interquartile Range); Statistical comparison was performed using + Mann Whitney test (P-value >0.05, Non-significant (NS); P-value< 0.01: highly significant (HS)).

**Table 5:** Linear Regression Model for Serum iron (μg/dL)

	Parameters statistics				Model statistics	
Factors	β	SE	p	95% CI	R2	p
Constant	87.25	5.66	0.001	75.96–98.54	0.276	0.001
H63D mutation	-13.34	9.6	0.169	-32.48–5.80		
Chronic HCV	15.23	6.59	0.024	2.08–28.38		
Interaction between H63D mutation and Chronic HCV	33.91	12.53	0.009	8.93–58.90		

Total cases, 100; β, Regression coefficient; SE, Standard error; CI, Confidence interval; R_2_, coefficient of determination.

## Discussion

*HFE* mutations are important gene variations in heredity haemochromatosis and a widespread autosomal recessive disorder correlated with iron excess in Caucasians. Hence, the role of HFE mutations in patients with HCV infection has been of great interest. Some studies were conducted to evaluate the correlations between HFE mutations, overload of hepatic iron and development of disease in CHC. Though, the impacts of HFE mutations on hepatic iron concentration and disease incidence remain elusive in Egyptian population (Ishizu et al., 2012). While, there is indication that a mild to severe iron overload is prevalent in patients with chronic HCV liver disease (Corengia et al., 2005), the importance of these findings with respect to liver injury pathogenesis and treatment relevance remains unclear (Bassett, 2007). Serum iron stores are often augmented in chronically infected patients with hepatitis C (Hasan and Brown, 2020). The current study aimed to study the HFE genetic mutation in addition to iron profile in HCV infected patients with Egyptian ancestry.

Current data revealed remarkable elevation in the concentration of serum total iron, serum ferritin and total iron binding capacity (TIBC) in chronic hepatitis C infected groups relative to the healthy group (P value <0.05). This was in accordance with Valenti et al who observed that iron related genes affect iron excess in patients with CHC, through study conducted on 143 chronic HCV patients (Valenti et al., 2007). Referring to Silva et al demonstrated that excess of hepatic iron was noted in patients with persistent HCV infection (Silva et al., 2005).

Martinelli et al., (2000) showed association between excessive accumulation of iron in the liver and accelerated fibrosis and cirrhosis in chronic HCV patients carrying HFE mutations (Cys282Tyr and His 63 Asp). Consequently, HFE mutations may be identified as crucial causes of co-morbidity in persistent HCV infection (Eisenbach et al., 2004). Several studies have shown that the prevalence of homozygosity of C282Y in HH patients in the Mediterranean basin was substantially lower than in northern Europe. While, Roth et al. and others showed that C282Y mutation is not existed in Algeria, Ethiopia, and Senegal (Roth et al., 1997). Moreover, Floreani et al., (2007) noticed that 2 % of their Italian population had C282Y mutations; they were both heterozygous. In Northern Italy, Valenti et al., (2007) have reported the same finding of a 2 % prevalence. In accordance with these findings, our analysis showed the absence of C282Y variant (Heterozygotes or homozygotes) in the studied cohort. 

Lauret et al., (2002) observed that the frequency of the heterozygous mutation C282Y in patients with HCC was substantially higher than those without HCC. This can be clarified by the geographical distribution of C282Y, which was observed to be missing in the African and Asian communities and limited to the northern European population (Acton et al., 2006). Martinelli et al reported that the incidence of H63D mutation was 23.7% in Brazil (Martinelli et al., 2000), while Valenti and Pelusi (2017) reported a 25 % prevalence of the same mutation in Italy. However, the incidence rate of H63D mutation has been declined in other geographical regions to be (13.98% in India) (Dhillon et al., 2007), (9.8% in Korea) (Lee et al., 2010) and (5.6 % in Japan) (Ishizu et al., 2012). Our results revealed that the prevalence of H63D mutation in our cohort was 23% and the distribution of the H63D homozygosity and heterozygosity in the control group was 4% and 32% respectively.

Several studies have indicated that the incidence of HFE gene mutations was higher in cirrhotic HCC patients relative to those without HCC (Lauret et al., 2002; Gharib et al., 2011). Gharib et al through a study of 300 Egyptian patients (100 cirrhotic patients, 100 HCC patients and 100 healthy subjects), found that H63D mutation D allele carriers were substantially more likely to develop HCC (Gharib et al., 2011). Kelley et al., (2014) reported a strong correlation between H63D homozygosity and the high transferring saturation compared to the wild genotype.

Dhillon et al., (2007) stated that even in the homozygous setting, the H63D mutation is not associated with iron overload. Our finding revealed that the H63D heterozygosity is significantly associated with the iron deposition in the cirrhotic patients without HCC development. While the correlation of H63D mutation and the iron deposition didn’t display statistical importance in cirrhotic patients with HCC. The later finding is in accordance with finding reported by Boige et al., (2003) who suggested that the presence of C282Y and H63D mutations did not seem to be correlated with an increased risk of HCC in cirrhosis patients, through research of 133 cirrhotic patients with and 100 cirrhotic patients without HCC. In addition, Ishizu et al., (2012) through study of 251 Japanese CHC patients, the frequencies of H63D mutation in the *HFE* gene have no impact on the clinical features of chronic hepatitis C infection.

Some researchers concur that polymorphisms of the *HFE* gene play a significant role in iron deposition, and therefore in the simultaneous progression of viral hepatitis. Although, it is not obvious whether increased iron indices are a phenomena arising from viral hepatitis, hepatic inflammation, and viral propagation, or whether concurrent *HFE*-gene polymorphisms may influence hepatitis-induced hepatic diseases (Erhardt et al., 2003). 

Our findings revealed that carriers of H63D mutant alleles among our groups did not provide substantial high levels giving the impression of the significance of HFE genotyping irrespective of iron studies as inferred by other authors (Hohler et al., 2000; Phatak et al., 2002) who stated that the C282Y mutation alone contributed to only a mild increase in iron accumulation in most patients and the existence of C282Y allele mutation even in the heterozygous state may be known as a risk for liver cirrhosis particularly in absence of further hepatopathic features. 

Debates over the interaction between HFE mutations and iron excess in chronic HCV patients definitely require several studies, including unique variables and mutations implicated in this mechanism as hepcidin, ferroportin, hemojuvelin, β-globin, and transferrin receptor-2 (TFR2) (Harigae, 2013).

In Conclusion, the present research highlights that there are considerable elevated serum iron markers in patients with chronic HCV infection. The frequency of C282Y mutation is not represented in the entire sample study, whereas the existence of H63D heterozygosity might contribute to the iron accumulation either in cirrhotic or non-cirrhotic without HCC patients. However, the HFE mutations didn’t seem to be associated with an elevated risk of HCC in cirrhosis patients. 

The limitations of the present study naturally include the lack of hepatic iron detection and relatively small sample size.

## Author Contribution Statement

Reham DAWOOD: Conceptualization, Project administration, Data curation, Funding acquisition, Formal analysis, Investigation, Methodology, Supervision, Validation, and Writing – original draft. Mai Abd El Meguid: Data curation, Methodology, and Validation, Writing – review & editing. Walied Elrobe: Resources, Software and Investigation. Ghada Salum: Data curation, Methodology, Investigation, Visualization. Naglaa Zayed: Resources, Validation and supervision. Sherief Mousa: Resources, Validation and supervision. Eman Medhat: Investigation, Validation and Supervision.

## References

[B1] Acton RT, Barton JC, Snively BM (2006). Geographic and racial/ethnic differences in HFE mutation frequencies in the Hemochromatosis and Iron Overload Screening (HEIRS) Study. Ethn Dis.

[B2] Bartolomei G, Cevik RE, Marcello A (2011). Modulation of hepatitis C virus replication by iron and hepcidin in Huh7 hepatocytes. J Gen Virol.

[B3] Bassett ML (2007). Iron and hepatitis C: beginning to make sense. J Gastroenterol Hepatol.

[B4] Blanc JF, De Ledinghen V, Bernard PH (2000). Increased incidence of HFE C282Y mutations in patients with iron overload and hepatocellular carcinoma developed in non-cirrhotic liver. J Hepatol.

[B5] Bloomer SA, Brown KE (2019). Iron-Induced Liver Injury: A Critical Reappraisal. Int J Mol Sci.

[B6] Bodmer JG, Parham P, Albert ED (1997). Putting a hold on “HLA-H’ The WHO Nomenclature Committee for Factors of the HLA System. Nat Genet.

[B7] Boige V, Castera L, de Roux N (2003). Lack of association between HFE gene mutations and hepatocellular carcinoma in patients with cirrhosis. Gut.

[B8] Britton RS, Ferrali M, Magiera CJ (1990). Increased prooxidant action of hepatic cytosolic low-molecular-weight iron in experimental iron overload. Hepatology.

[B9] Corengia C, Galimberti S, Bovo G (2005). Iron accumulation in chronic hepatitis C: relation of hepatic iron distribution, HFE genotype, and disease course. Am J Clin Pathol.

[B10] Daher RT, Khalik RN, Hoteit RM (2011). The use of a reverse hybridization strip assay for the study of hemochromatosis-associated gene mutations in Lebanon. Genet Test Mol Biomarkers.

[B11] Dhillon BK, Das R, Garewal G (2007). Frequency of primary iron overload and HFE gene mutations (C282Y, H63D and S65C) in chronic liver disease patients in north India. World J Gastroenterol.

[B12] Drakesmith H, Prentice A (2008). Viral infection and iron metabolism. Nat Rev Microbiol.

[B13] Eisenbach C, Gehrke SG, Stremmel W (2004). Iron, the HFE gene, and hepatitis C. Clin Liver Dis.

[B14] Erhardt A, Maschner-Olberg A, Mellenthin C (2003). HFE mutations and chronic hepatitis C: H63D and C282Y heterozygosity are independent risk factors for liver fibrosis and cirrhosis. J Hepatol.

[B15] Faul F, Erdfelder E, Lang AG (2007). G*Power 3: a flexible statistical power analysis program for the social, behavioral, and biomedical sciences. Behav Res Methods.

[B16] Feder JN, Gnirke A, Thomas W (1996). A novel MHC class I-like gene is mutated in patients with hereditary haemochromatosis. Nat Genet.

[B17] Floreani A, Rosa Rizzotto E, Basso D (2007). An open population screening study for HFE gene major mutations proves the low prevalence of C282Y mutation in Central Italy. Aliment Pharmacol Ther.

[B18] Fracanzani AL, Conte D, Fraquelli M (2001). Increased cancer risk in a cohort of 230 patients with hereditary hemochromatosis in comparison to matched control patients with non-iron-related chronic liver disease. Hepatology.

[B19] Gao J, Chen J, Kramer M (2009). Interaction of the hereditary hemochromatosis protein HFE with transferrin receptor 2 is required for transferrin-induced hepcidin expression. Cell Metab.

[B20] Georgopoulou U, Dimitriadis A, Foka P (2014). Hepcidin and the iron enigma in HCV infection. Virulence.

[B21] Gharib AF, Karam RA, Pasha HF (2011). Polymorphisms of hemochromatosis, and alpha-1 antitrypsin genes in Egyptian HCV patients with and without hepatocellular carcinoma. Gene.

[B22] Girelli D, Pasino M, Goodnough JB (2009). Reduced serum hepcidin levels in patients with chronic hepatitis C. J Hepatol.

[B23] Gochee PA, Powell LW, Cullen DJ (2002). A population-based study of the biochemical and clinical expression of the H63D hemochromatosis mutation. Gastroenterology.

[B24] Hanson EH, Imperatore G, Burke W (2001). HFE gene and hereditary hemochromatosis: a HuGE review. Human Genome Epidemiology. Am J Epidemiol.

[B25] Harigae H (2013). Cutting-edge of medicine; iron metabolism--recent findings. Nihon Naika Gakkai Zasshi.

[B26] Hasan Y, Brown K (2020). Viral eradication restores normal iron status in chronic hepatitis C patients with abnormal iron studies. Ann Hepatol.

[B27] Hohler T, Leininger S, Kohler HH (2000). Heterozygosity for the hemochromatosis gene in liver diseases--prevalence and effects on liver histology. Liver.

[B28] Horl WH, Schmidt A (2014). Low hepcidin triggers hepatic iron accumulation in patients with hepatitis C. Nephrol Dial Transplant.

[B29] Ishizu Y, Katano Y, Honda T (2012). Clinical impact of HFE mutations in Japanese patients with chronic hepatitis C. J Gastroenterol Hepatol.

[B30] Jeffrey GP, Chakrabarti S, Hegele RA (1999). Polymorphism in intron 4 of HFE may cause overestimation of C282Y homozygote prevalence in haemochromatosis. Nat Genet.

[B31] Kelley M, Joshi N, Xie Y (2014). Iron overload is rare in patients homozygous for the H63D mutation. Can J Gastroenterol Hepatol.

[B32] Lauret E, Rodriguez M, Gonzalez S (2002). HFE gene mutations in alcoholic and virus-related cirrhotic patients with hepatocellular carcinoma. Am J Gastroenterol.

[B33] Lee SH, Jeong SH, Lee D (2010). An epidemiologic study on the incidence and significance of HFE mutations in a Korean cohort with nonalcoholic fatty liver disease. J Clin Gastroenterol.

[B34] Martinelli AL, Franco RF, Villanova MG (2000). Are haemochromatosis mutations related to the severity of liver disease in hepatitis C virus infection?. Acta Haematol.

[B35] Masavuli MG, Wijesundara DK, Underwood A (2019). A hepatitis C virus DNA vaccine encoding a secreted, oligomerized form of envelope proteins is highly immunogenic and elicits neutralizing antibodies in vaccinated mice. Front Immunol.

[B36] Moysés CB, Moreira ES, Asprino PF (2008). Simultaneous detection of the C282Y, H63D and S65C mutations in the hemochromatosis gene using quenched-FRET real-time PCR. Brazil J Med Biol Res.

[B37] Nishina S, Hino K, Korenaga M (2008). Hepatitis C virus-induced reactive oxygen species raise hepatic iron level in mice by reducing hepcidin transcription. Gastroenterology.

[B38] Phatak PD, Ryan DH, Cappuccio J (2002). Prevalence and penetrance of HFE mutations in 4865 unselected primary care patients. Blood Cells Mol Dis.

[B39] Pietrangelo A (2006). Hereditary hemochromatosis. Annu Rev Nutr.

[B42] Roth M, Giraldo P, Hariti G (1997). Absence of the hemochromatosis gene Cys282Tyr mutation in three ethnic groups from Algeria (Mzab), Ethiopia, and Senegal. Immunogenetics.

[B43] Sendi H, Mehrab-Mohseni M (2012). HFE gene mutations in cryptogenic cirrhosis patients. Hepat Mon.

[B44] Silva IS, Perez RM, Oliveira PV (2005). Iron overload in patients with chronic hepatitis C virus infection: clinical and histological study. J Gastroenterol Hepatol.

[B45] Thorén F, Romero A, Lindh M (2004). A hepatitis C virus-encoded, nonstructural protein (NS3) triggers dysfunction and apoptosis in lymphocytes: role of NADPH oxidase-derived oxygen radicals. J Leukoc Biol.

[B46] Vagu C, Sultana C, Ruta S (2013). Serum iron markers in patients with chronic hepatitis C infection. Hepat Mon.

[B47] Valenti L, Pelusi S (2017). HFE mutations and iron in hemodialysis patients. Hemodial Int.

[B48] Valenti L, Pulixi EA, Arosio P (2007). Relative contribution of iron genes, dysmetabolism and hepatitis C virus (HCV) in the pathogenesis of altered iron regulation in HCV chronic hepatitis. Haematologica.

[B49] Van Bokhoven MA, van Deursen CT, Swinkels DW (2011). Diagnosis and management of hereditary haemochromatosis. BMJ.

[B50] Vela D (2018). Low hepcidin in liver fibrosis and cirrhosis; a tale of progressive disorder and a case for a new biochemical marker. Mol Med.

[B51] Ye Q, Qian BX, Yin WL (2016). Association between the HFE C282Y, H63D polymorphisms and the risks of non-alcoholic fatty liver disease, liver cirrhosis and hepatocellular carcinoma: An Updated Systematic Review and Meta-Analysis of 5,758 Cases and 14,741 Controls. PLoS One.

